# Clinical and Electrocardiographic Findings for Predicting the Severity of Pulmonary Valve Stenosis in Dogs

**DOI:** 10.3390/vetsci9020061

**Published:** 2022-02-01

**Authors:** Martina Bini, Tommaso Vezzosi, Maria Josefa Fernández Del Palacio, Jesús Talavera, Valentina Patata, Federica Marchesotti, Oriol Domenech

**Affiliations:** 1 Anicura Istituto Veterinario Novara, Strada Provinciale 9, Granozzo con Monticello, 28060 Novara, Italy; tommaso.vezzosi@anicura.it (T.V.); valentina.patata@anicura.it (V.P.); federica.marchesotti@anicura.it (F.M.); oriol.domenech@anicura.it (O.D.); 2 Department of Veterinary Sciences, University of Pisa, Via Livornese, San Piero a Grado, 56122 Pisa, Italy; 3 Department of Animal Medicine and Surgery, Veterinary Teaching Hospital, 30100 Murcia, Spain; mjfp@um.es (M.J.F.D.P.); talavera@um.es (J.T.)

**Keywords:** cardiology, electrocardiography, heart murmur, mean electrical axis, QRS complex

## Abstract

Pulmonary valve stenosis (PS) in dogs is usually suspected due to the presence of a heart murmur and clinical signs. Echocardiography is needed to confirm the diagnosis and define the severity of PS. This retrospective study evaluated the utility of clinical and electrocardiographic (ECG) findings in the prediction of PS severity. Data regarding heart murmur and ECG analysis were gathered. Ninety-seven dogs with PS were included. A murmur grade ≥IV/VI was predictive of severe PS (area under curve (AUC) = 0.71; sensitivity (Se) = 95%; specificity (Sp) = 33%; *p* = 0.003). In lead II, P wave amplitude >0.35 mV (AUC = 0.67; Se = 31%; Sp = 100%; *p* = 0.038), Q wave < 0.15 mV (AUC = 0.70; Se = 70%; Sp = 59%; *p* = 0.0015), R wave < 0.87 mV (AUC = 0.66; Se = 67%; Sp = 69%; *p* = 0.006), and S wave > 0.37 mV (AUC = 0.80; Se = 72%; Sp = 85%; *p* < 0.0001) were predictive of severe PS. The extent of right deviation of the mean electrical axis of the QRS complex was correlated with the pulmonary pressure gradient (r = 0.648; *p* < 0.0001). In conclusion, a systolic murmur with intensity ≥IV/VI, a P wave amplitude >0.35 mV, low amplitude of Q and R waves, deep S waves in lead II, and right axis deviation of the QRS complex in a young dog are predictive of severe PS.

## 1. Introduction

Pulmonary valve stenosis (PS) is one of the most common congenital heart diseases in dogs. PS represents 31–34% of congenital heart diseases in dogs [1,2,3]. First opinion veterinarians often suspect PS following the detection of a left basilar systolic murmur in a young dog [4,5]. Less frequently, affected dogs may show clinical signs such as exercise intolerance, syncope, or right-sided congestive heart failure [6]. Although the aforementioned clinical findings are highly suggestive of PS in a young dog, echocardiographic evaluation remains the gold standard for the diagnosis and the classification of PS; however, it requires specialized equipment and a trained cardiologist. Moreover, assessing the severity of the stenosis is decisive for the therapeutic management [6].

Pulmonary balloon valvuloplasty is considered the elective treatment for isolated severe PS [4,6,7,8]. In this context, easily obtained and affordable complementary diagnostic tests, such as an electrocardiogram (ECG), may be useful indirect indicators of heart disease. Different ECG variables have been correlated with right ventricular (RV) enlargement.

To the best of our knowledge, no previous studies have investigated the possible correlation between ECG findings and PS severity determined by Doppler echocardiography. This would enable first opinion veterinarians to integrate this information within clinical examination signs, thus helping to identify dogs possibly suffering from severe PS. The use of a systematic clinical and diagnostic approach aimed at early identification of dogs with severe PS assumes great importance, not only to apply a suitable medical and/or surgical workup and formulate a correct prognosis but also to prevent any legal dispute [9].

The aim of this study was therefore to evaluate the utility of clinical and ECG findings in the prediction of severe PS.

## 2. Materials and Methods

This was a retrospective observational study; therefore, no institutional animal care and use approval or client consent were sought. Medical records of dogs referred for cardiovascular investigation at the Cardiology Department of the Anicura Istituto Veterinario Novara (Italy) between December 2011 and December 2019 and at the Cardiology Service of Veterinary Teaching Hospital of the University of Murcia (Spain) between January 1996 and January 2008 were reviewed for all cases of PS that had undergone a complete echocardiographic examination and a surface 6-lead ECG on the same day. Dogs with concomitant congenital or acquired heart disease other than PS were excluded. Dogs with trivial or mild regurgitation of the tricuspid, mitral, and aortic valves were not excluded provided that the valve apparatus was normal.

In all dogs, PS was diagnosed by echocardiography by a board-certified veterinary cardiologist or a cardiology resident under the supervision of a board-certified veterinary cardiologist.

Pulmonary valve stenosis morphology and severity were classified as previously reported [10]. Doppler-derived peak transvalvular pulmonary pressure gradient (PG) was used to define the severity of PS: the stenosis was classified as mild when the PG was less than 50 mmHg, moderate when between 50 and 80 mmHg, and severe when the PG was greater than 80 mmHg [10,11].

For each dog included, data collected from the medical records comprised patient breed, age at diagnosis, sex, body weight, and clinical signs compatible with right-sided congestive heart failure. The presence of right-sided congestive heart failure was based on the presence of ascites associated with jugular venous distension and a subjectively dilated caudal vena cava. Particular attention was paid to the presence of a heart murmur, the point of maximal intensity, and murmur grade, according to the six-level scheme [12].

### 2.1. Electrocardiographic Examination

A standard six-lead ECG recording was performed in the same manner for all patients. Unsedated dogs were gently restrained in right lateral recumbence with the owner present and flattened alligator electrodes were attached to the forelimbs and hindlimbs as previously recommended [13,14]. The ECG tracings were obtained using two different electrocardiographs (MAC600, General Electric; Siemens Megacart R, Electromedical System Division), at a paper speed of 50 mm/s or 25 mm/s and a vertical ECG calibration of 5 mm/mV, 10 mm/mV, or 20 mm/mV. The electrocardiographic units were set with a sampling frequency of 1000 Hz for acquisition, a 100 Hz low-pass filter and a 0.3–0.5 Hz high-pass filter to eliminate oscillations of the isoelectric line due to breathing [13]. Tracings were reviewed by two operators (M.B. and M.J.F.P.). For each of the tracings, the following ECG variables were assessed: mean heart rate expressed in beats per minute (bpm); P wave amplitude expressed in millivolts (mV); QRS complex duration expressed in milliseconds (ms); Q, R, and S wave amplitude (mV); and mean electrical axis (MEA) of the QRS complex expressed in degrees (°). The presence of complete right bundle branch block, atrial, or ventricular arrhythmias was noted.

The duration and amplitude of the ECG waveforms were manually measured in lead II. A P wave amplitude less than 0.4 mV was considered normal [13,15,16]. The morphology of the QRS complex was described using a capital letter if the amplitude of the wave was greater than 0.5 mV (“Q”, “R”, or “S”), or a lower-case letter (“q”, “r”, or “s”) if the amplitude was less than 0.5 mV [13]. Normal QRS complex duration was considered if less than 70 ms, and normal R wave amplitude if less than 2.5 mV in small breed dogs (<20 kg), and less than 3 mV in large breed dogs (>20 kg) [13,14,15,16].

The MEA of the QRS complex in the frontal plane was calculated using the isoelectric method and was considered within normal limits between +40° and +100° [13,15,16]. Complete right bundle branch block was defined as a QRS complex duration exceeding 80 ms due to a wide S wave and a right shift of the MEA of the QRS complex up to −110° [13].

In order to evaluate a possible correlation between the severity of PS and MEA of the QRS complex, a corrected MEA was created. The aim was to obtain only positive values of the MEA, proportional to the extent of deviation in the case of negative values. The following formula was used:

corrected MEA = 360 + x

given x as the negative value of the MEA. Using this calculation, it was thus possible to obtain only increasing positive values in the third quadrant (from ± 180° to −90°) and in the fourth quadrant (from −90° to 0°) from + 180° to + 360°. For example, a MEA of the QRS equal to −150° corresponds to a corrected MEA of +210°, using the above-mentioned calculation: 360–150.

### 2.2. Statistical Analysis

Statistical analyses were performed using Prism 5 (GraphPad software, San Diego, CA, USA) and SPSS statistics 22 (IBM, Armonk, NY, USA). Descriptive statistics were generated, and the Shapiro–Wilk test was used for normality testing of all data. The non-normally distributed variables were reported as the median and range, those normally distributed were reported as the mean ± standard deviation. A non-parametric Kruskal–Wallis test or a Mann–Whitney–Wilcoxon test was applied to analyze differences in clinical and electrocardiographic variables between the groups (dogs with mild, moderate, and severe PS). A receiver operating characteristics (ROC) analysis was performed to calculate the sensitivity (Se) and specificity (Sp) of the heart murmur grade and ECG parameters in the prediction of PS severity. An area under the curve (AUC) > 0.7 indicated that the diagnostic test had a good diagnostic accuracy [17]. The Youden index was used to identify the best cut-off value of the clinical and electrocardiographic variables in order to discriminate between dogs affected by mild-to-moderate PS and severe PS. Correlations between Q, R, and S wave amplitude, corrected MEA of the QRS complex, and the echocardiographic severity of PS were tested. Correlations were considered high when r > 0.7, moderate if r was between 0.7 and 0.5, weak if r was between 0.5 and 0.3, and no correlation if r < 0.3 [18,19]. A value of *p* < 0.05 was considered statistically significant.

## 3. Results

A total of 97 dogs of different breeds affected by PS were enrolled in the study. Breeds included were French Bulldog (n = 20), English Bulldog (n = 13), mixed-breed (n = 9), Boxer (n = 8), Yorkshire Terrier (n = 5), Bullmastiff (n = 4), English Cocker Spaniel (n = 4), American Staffordshire Terrier (n = 3), German Shepherd (n = 3), West Highland White Terrier (n = 3), Maltese (n = 2), Mastiff (n = 2), Miniature Pinscher (n = 2), Pomeranian (n = 2), Poodle (n = 2), and one of each of the following breeds: Affenpinscher, Australian Shepherd, Border Collie, Breton, Cane Corso, Chihuahua, Dobermann, Hannover Hound, Jack Russell Terrier, Labrador, Pointer, Rhodesian Ridgeback, Spitz, Whippet, and White Swiss Shepherd Dog. There were 56 males (58%) and 41 females (42%). The median age was 1.3 years (range 0.2–13 years).

Pulmonary valve stenosis was mild in 15/97 dogs (15%), moderate in 24/97 dogs (25%), and severe in 58/97 dogs (60%). Median PG was 96 mmHg (12–250 mmHg) in the overall population. The median peak PG of the group with mild PS was 40 mmHg (12–49 mmHg), 64.5 mmHg (51–79 mmHg) in the moderate PS group, and 133 mmHg (80–250 mmHg) in dogs with severe PS (Table 1).

### 3.1. Clinical Signs

A total of 28 out of 97 (29%) dogs showed clinical signs at the time of presentation. Of those, 10/28 (36%) presented with jugular vein distension and ascites compatible with right-sided congestive heart failure, 13/28 (47%) had syncope, and 4/28 (14%) had exercise intolerance. Twenty-three (82%) of the symptomatic dogs had severe PS, and clinical signs were significantly more frequent in the severe group than in the mild-to-moderate group (*p* = 0.0001; Se = 40%; Sp = 95%).

Pulse quality was good in the majority of dogs with the exception of 13/97 (13%) dogs who had weak femoral pulse. All of them presented severe PS and 7/13 (54%) had right-sided congestive heart failure.

**Figure 1:**
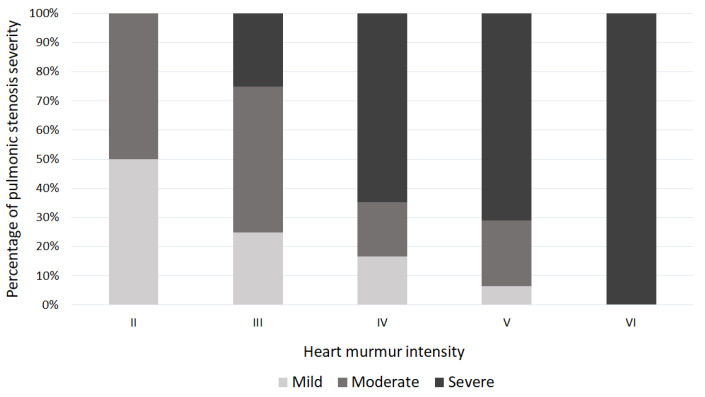
Distribution of pulmonary valve stenosis severity across different heart murmur intensities.

A left basilar systolic murmur was found in all dogs, with a median grade of IV/VI (range II/VI–VI/VI). In the study sample, 81/97 dogs (83%) had a murmur intensity equal or higher than IV/VI, and 55 (68%) had severe PS. On the other hand, among dogs with II/VI or III/VI murmurs, 13/16 (81%) presented mild or moderate PS (Figure 1). The presence of a systolic murmur with a murmur grade ≥ IV/VI was predictive of severe PS with a good diagnostic accuracy (area under curve (AUC) = 0.71; Se = 95%; Sp = 33%; *p* = 0.003).

### 3.2. Electrocardiography

Table 1 contains the summary data of the ECG findings divided according to PS severity. In the study sample, 9/97 dogs (9%) had a *p* wave amplitude of >0.4 mV, and all these dogs had severe PS. The P wave amplitude showed a low sensitivity but very high specificity for the detection of severe PS, with an optimal cut-off of >0.35 mV in lead II for the prediction of severe PS (AUC = 0.67; Se = 31%; Sp = 100%; *p* = 0.038).

The QRS complex duration was increased in 6/97 (6%) dogs, and all of these dogs had severe PS (Se = 10%; Sp = 100%). Interestingly, all the dogs with a prolonged duration of QRS complex showed a duration greater than 80 ms associated with a right shift of the MEA compatible with complete right bundle branch block. However, there was no significant difference in the percentage of dogs with a right bundle branch block comparing dogs with severe PS to dogs with mild-to-moderate PS (*p* = 0.29).

**Figure 2:**
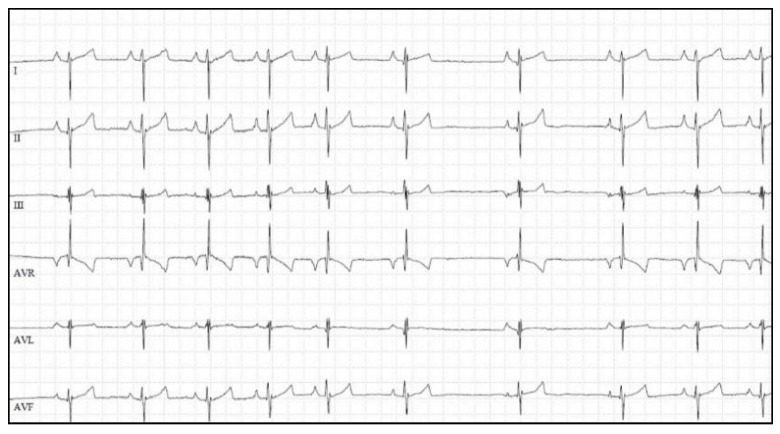
Six-lead electrocardiogram of a dog with severe pulmonary valve stenosis (peak transvalvular pulmonary pressure gradient: 114 mmHg) demonstrating sinus arrhythmia with a mean heart rate of 120 bpm, a P wave amplitude of 0.35 mV, a RS morphology of QRS complex in lead II, and right deviation of the mean electrical axis (−135°). Paper speed = 50 mm/s; 10 mm/mV.

**Figure 3:**
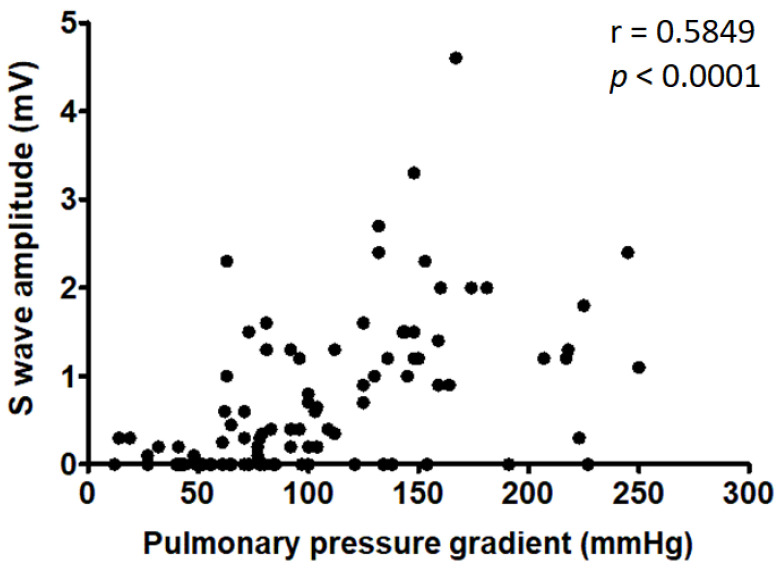
Positive correlation between S wave amplitude in lead II and pulmonary pressure gradient. Black dots are representing the study population.

Analysis of the QRS complex in lead II revealed that the most represented QRS morphologies were RS (26/97; 27%) and qR (17/97; 17%) (Figure 2). There was a relationship between QRS morphology and PS severity, with RS morphology being more frequent in dogs with severe PS and with qR morphology being more prevalent in the mild-to-moderate group (*p* = 0.0004). The amplitude of the S wave showed a moderate positive correlation with the PG (r = 0.585, *p* < 0.0001) (Figure 3). The amplitude of Q and R waves showed a weak negative correlation with the PG (Q wave: r = –0.396, *p* < 0.0001; R wave: r = −0.384, *p* = 0.0001). The optimal cut-off of the Q wave amplitude in differentiating dogs with severe PS from dogs with mild-to-moderate PS was < 0.15 mV with a good diagnostic accuracy (AUC = 0.70; Se = 70%; Sp = 59%; *p* = 0.0015). An R wave amplitude of < 0.87 mV identified dogs with severe PS with a sufficient diagnostic accuracy (AUC = 0.66; Se = 67%; Sp = 69%; *p* = 0.006). The optimal cut-off of S wave amplitude for the prediction of severe PS was > 0.37 mV (AUC = 0.80; Se = 72%; Sp = 85%; *p* < 0.0001).

**Figure 4:**
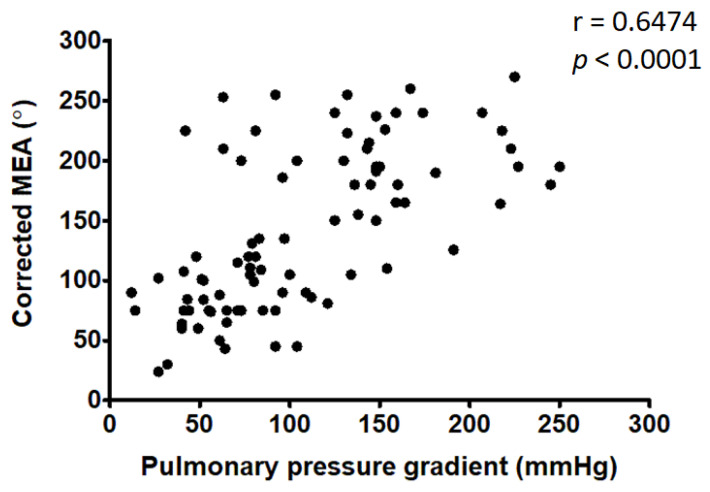
Positive correlation between the corrected MEA and pulmonary pressure gradient. Black dots are representing the study population.

Fifty-six dogs (58%) showed a right shift of the MEA of the QRS complex, and the majority of these (45/56, 80%) had severe PS. A corrected MEA cut-off of > 133° had a very good diagnostic accuracy for the prediction of severe PS (AUC = 0.82; Se = 70%; Sp = 89%; *p* < 0.0001). The corrected MEA showed a moderate positive linear correlation with the PG (r = 0.648, *p* < 0.0001) (Figure 4).

There were no significant differences in the mean heart rate of dogs with varying degrees of severity and in the percentage of dogs with atrial arrhythmias and ventricular arrhythmias comparing dogs with mild-to-moderate PS to dogs with severe PS (Table 1).

## 4. Discussion

To the best of our knowledge, this is the first study of dogs with PS that has compared clinical and electrocardiographic data with the PG. Our results suggest that murmur intensity and ECG findings can be useful in the clinical prediction of PS severity in dogs.

It is generally recommended, both in human and veterinary medicine, that severe PS should undergo both medical and interventional treatment even in asymptomatic cases because of the high risk of progressive dilatation and decreased systolic performance of the RV, possibly resulting in right heart failure and death [6,20,21,22,23,24,25].

Dogs with severe PS have a guarded long-term prognosis, and early detection of the disease is crucial to identify those that could benefit from pulmonary balloon valvuloplasty [5,6,7,26]. First opinion veterinarians play a central role in the early detection of dogs with congenital heart disease, considering that most present with a heart murmur. Although echocardiography is the non-invasive gold standard for PS diagnosis, epidemiology, clinical history, physical examination, and easy to acquire and affordable complementary diagnostic tests such as 6-lead ECG can help in the suspicion of the underlying disease and possibly suggest its severity.

The epidemiological characteristics of our sample were in accordance with previous studies. The main breeds affected were brachycephalic, mostly represented by the French Bulldog and English Bulldog, in agreement with previous literature [3,6,7]. The median age at first presentation, 1.3 years in our study, was comparable with a previous study on 30 dogs with PS and a mean age of 16.31 ± 5.3 months [7].

The percentage of dogs with clinical signs at first examination, 29%, was comparable with other studies [6,26]. Overt clinical signs were more frequent in dogs with severe PS and this was in agreement with previous studies in human and veterinary medicine that have reported an association between the presence of clinical signs and PS severity [8,27,28,29]. Furthermore, previous literature also demonstrated that patients with clinical signs are at greater risk of cardiac death [6,8]. Thus, the identification of clinical and electrocardiographic abnormalities that allow early recognition of severe PS and intervention before the onset of clinical signs plays an essential role in order to provide a good life span in the canine population.

The vast majority of dogs affected by PS present with an incidentally detected murmur. Our study sample supports this since all dogs included had a systolic murmur with the point of maximum intensity at the left base. Heart murmur intensity tends to be proportionally related to the severity in dogs with PS and sub-aortic stenosis [30,31]. In our study, most dogs (68%) with a loud or palpable murmur (i.e., IV to VI/VI) had severe PS, and all cases with a mild murmur (i.e., I to II/VI) had mild-to-moderate PS. In our sample, 32% of the dogs with loud or palpable murmur had mild or moderate PS. Caivano et al. 2017 found that 20% of dogs with a loud or palpable murmur suffered from mild-to-moderate stenosis [30]. Similarly, Rishniw et al. 2019 showed a similar percentage of dogs with loud or palpable murmur and mild-to-moderate stenosis (20%) [31]. In our population, the percentage of dogs with a murmur grade of ≥ IV/VI with mild-to-moderate stenosis was slightly higher than the aforementioned studies. This difference could be attributable to the different inclusion criteria. Caivano and Rishniw included dogs suffering from PS and sub-aortic stenosis, in contrast with our study that only included dogs with PS. The hypothesis that murmur intensity may be influenced by the underlying disease at the same PG could be supported by the evidence that in the study by Caivano et al. 2017 of the dogs with palpable murmurs and mild-to-moderate stenosis, the six lowest gradients were all found in dogs with PS. Moreover, all the dogs with mild stenosis and palpable murmurs had PS [30]. Whether this reflects a difference in murmur intensity and PG between PS and sub-aortic stenosis is unclear, but it could be a possible explanation for the higher percentage of dogs with loud or palpable murmur and mild-to-moderate PS found in our study. In any case, a systolic murmur grade of ≥ IV/VI should alert the clinician to the probability of severe stenosis, considering the good diagnostic accuracy that we found. However, given the low specificity, it does not make it possible to definitely rule out the presence of a mild-to-moderate PS.

In our study most of patients presented normal femoral pulse quality, as previously reported [11]. Only 13% of dogs from our study presented weak femoral pulse, and all of them had severe PS. Thus, the presence of a weak pulse in dogs with PS may give clinical information about PS severity.

Specific electrocardiographic criteria for the detection of right heart enlargement have been described both in humans and in dogs. In infants with mild PS, the ECG may be normal, although frequently there is delayed RV conduction. On the other hand, more severe stenosis is associated with right axis deviation, RV hypertrophy pattern, and increased amplitude of the P waves [27].

The ECG criteria intended to detect RV enlargement in dogs still in use today come from a study published in 1971 by Hill, in which deep S waves in leads I, II, III, and aVF represent the most important finding. The deep S waves result in a MEA shifting to the right and cranially, with more severe ECG changes evidenced in dogs with congenital right heart disease [32]. In line with this, we found a positive correlation between S wave amplitude in lead II and PS severity. The increases in S wave voltage related to RV hypertrophy have been extensively described in dogs [15,28,33]. Hill reported a S wave amplitude of >0.35 mV in lead II in 50% of dogs with RV enlargement due to congenital or acquired disease. The cut-off of >0.35 mV for S wave amplitude in lead II is still considered one of the main ECG criteria for the diagnosis of RV enlargement in dogs [34]. We found statistical differences for the amplitude of S waves between mild-to-moderate PS and severe PS, suggesting an association between increased S wave amplitude and PG. Consequently, increased S wave amplitude—and particularly values >0.37 mV in a young dog of a predisposed breed presenting a left basal systolic murmur—might be indicative, not only of right heart enlargement but also of a possible diagnosis of severe PS.

To our knowledge, this is the first study to describe the variations in Q and R wave amplitudes in lead II in dogs with PS. Interestingly, the Q wave decreases its amplitude as the PG increases, and it is often absent in tracings recorded from dogs with severe PS. We identified a cut-off of 0.15 mV, below which it is possible to suspect severe PS with good diagnostic accuracy. However, this result may be of little clinical utility as the Q wave may often be absent or of low amplitude in the lead II of healthy dogs’ ECGs [35]. Similarly, an R wave amplitude <0.87 mV can be frequently detected in healthy dogs, especially in dogs with a broad chest (e.g., French Bulldogs or English Bulldogs), which are the breeds most affected by PS [3,6,7]. The relationship between the Q and R wave amplitudes in lead II and PG, although statistically significant, is therefore not biologically significant, which lacks practical utility in routine daily clinical practice.

Our study has confirmed the direct correlation between the extent of right deviation of the MEA and the pulmonary PG, in agreement with a number of previous human and veterinary studies [15,32,36,37]. The close association between RV enlargement secondary to severe PS and MEA is detectable immediately after birth in dogs. Trautvetter et al. observed that modal QRS vectors of pups with severe PS could be used to recognize pathologic RV hypertrophy, despite being superimposed on the normally present “physiologic hypertrophy” of the pup [38]. However, ours is the first study to identify a precise corrected MEA cut-off of 133°, beyond which it is possible to detect severe PS with very good diagnostic accuracy.

Although in the majority of dogs these ECG parameters were normal, analysis of the P wave and the QRS complex showed that a P wave amplitude in lead II > 0.35 mV and an increased QRS complex duration have low sensitivity but a specificity of 100% in predicting severe PS. Furthermore, all dogs that presented an increased QRS complex duration showed a concomitant right shift of the MEA consistent with a complete right bundle branch block. These findings could be clinically significant since the 100% specificity ensures that the presence of severe PS would definitely lead to a positive test result. The detection of a P wave amplitude in lead II > 0.35 mV or a complete right bundle branch block in a young dog where PS is suspected indicates that the stenosis is severe. Previous studies in human and veterinary medicine have reported an increased P wave amplitude and complete right bundle branch block as infrequent ECG findings associated with PS [11,36]. However, to the best of our knowledge, this is the first study in veterinary medicine to describe the incidence of these ECG changes in dogs with PS and their potential clinical use in predicting the severe form of this disease.

Our study has some limitations. Due to the retrospective design, the number of dogs included with mild and moderate PS was lower than the number of dogs with severe PS. These unbalanced group sizes could thus have reduced the statistical power to detect differences between some variables or between dogs with mild PS and dogs with moderate PS. However, the clinical impact of this limitation is probably of little significance since moderate PS is generally associated with a favorable long-term prognosis with a limited number of cardiac-related deaths [6]. Moreover, mild and moderate PS are less frequent in clinical practice, particularly in referral clinics, as already identified in previous studies [5,6,25]. Thus, further studies with more balanced groups of dogs with mild, moderate, and severe PS are still recommended.

Ventricular function was not assessed in the dogs in our study. It is known that in dogs with severe PS and RV systolic dysfunction, PG decreases, and the classification of PS severity may not be adequate in all cases [39]. However, of the total number of dogs included, only 10/97 (10%) showed clinical signs compatible with right heart failure, and all had a PG > 80 mmHg, thus making it unlikely that some dogs were misclassified.

We found no significant differences in the prevalence of atrial and ventricular arrhythmias when comparing mild-to-moderate PS and severe PS. However, it is not possible to show the real prevalence of such arrhythmias without Holter ECG monitoring. A resting ECG is typically recorded for several seconds to several minutes and is likely to miss or underestimate the intermittent arrhythmias. Despite this, human PS is not often associated with significant arrhythmias, particularly in those who have not undergone surgical palliation of the stenosis [40,41].

Information on nutritional status, such as Body Condition Score, was not available at the time of data collection. This is a limitation since the fatness or thinness of an individual can influence the morphology and amplitude of the electrocardiographic waves [13]. In particular, the identification and exclusion of obese dogs would have been recommendable since the interposition of fat between the heart and the surface electrodes may decrease the amplitude of R wave [13].

Lastly, precordial leads used to explore QRS complex morphology were not performed in most dogs in our study; thus, the diagnostic accuracy of the precordial lead system for the diagnosis of severe PS was not evaluated. This is a limitation, as studies both in human [36,42,43] and veterinary medicine [15,32,34] have described ECG abnormalities in precordial leads in dogs with RV hypertrophy and enlargement. Further studies are necessary to assess the diagnostic accuracy of precordial lead criteria in predicting severe PS in dogs.

## 5. Conclusions

In this study, a loud (≥IV/VI) left basilar systolic murmur, RS morphology of the QRS complex with deep S waves in lead II, and a corrected MEA of the QRS complex > 133° showed good diagnostic accuracy for the detection of severe PS in dogs. In addition, the presence of a P wave amplitude in lead II > 0.35 mV and of a complete right bundle branch block in addition to the previous clinical and ECG findings can be decisive in predicting severe PS. Definitive diagnosis and severity evaluation must always be established by a complete echocardiographic examination. Nevertheless, these criteria could help first opinion veterinarians to suspect a severe form of PS, thus necessitating an urgent echocardiographic evaluation in order to consider the best therapeutic choice.

## Figures and Tables

**Table 1:** Electrocardiographic findings in 97 dogs with pulmonary valve stenosis according to severity.

Electrocardiographic Findings	PS Severity			*p*-Value
	Mild (n = 15) PG 40 mmHg (12–49)	Moderate (n = 24) PG 64.5 mmHg (51–79)	Severe (n = 58) PG 133 mmHg (80–250)	
Heart rate (bpm)	129 (±31)	130 (±31)	126 (±36)	0.62
Rhythm	Sinus rhythm (n = 14) VPCs (n = 1)	Sinus rhythm (n = 22) APCs (n = 2)	Sinus rhythm (n = 50) APCs (n = 3) AF (n = 3) VPCs (n = 2)	0.33
P wave amplitude (mV)	0.2 (0.1–0.2)	0.2 (0.1–0.3)	0.3 (0.1–0.6)	0.03
QRS complex duration (ms)	49 (±12)	52 (±9)	51 (30–93)	0.56
Q wave amplitude (mV)	0.4 (±0.2)	0.1 (0–0.9)	0 (0–1.2)	0.0007
R wave amplitude (mV)	1.2 (±0.6)	1 (±0.5)	0.7 (0–2.7)	0.006
S wave amplitude (mV)	0 (0–0.3)	0.05 (0–2.3)	1 (0–4.6)	<0.0001
Corrected MEA of the QRS (°)	75 (24–225)	86 (43–253)	180 (45–270)	<0.0001

Abbreviations: AF, atrial fibrillation; APCs, atrial premature complexes; bpm, beats per minute; MEA, mean electrical axis; ms, milliseconds; mV, millivolt; PG, peak transvalvular pulmonary pressure gradient PS: pulmonic stenosis; VPCs, ventricular premature complexes; °, degrees. Note: Normally distributed data are presented as mean (± standard deviation), non-normally distributed data are presented as median (range). *p*-value represents the comparison of mild-to-moderate PS with severe PS. Values in bold denote statistical significance.

## Data Availability

The data presented in this study have not been published elsewhere, but are available on request from the corresponding author.
